# Cretaceous amniote integuments recorded through a taphonomic process unique to resins

**DOI:** 10.1038/s41598-020-76830-8

**Published:** 2020-11-16

**Authors:** Sergio Álvarez-Parra, Xavier Delclòs, Mónica M. Solórzano-Kraemer, Luis Alcalá, Enrique Peñalver

**Affiliations:** 1 Departament de Dinàmica de la Terra i de l’Oceà and Institut de Recerca de la Biodiversitat (IRBio), Facultat de Ciències de la Terra, Universitat de Barcelona, c/ Martí i Franquès S/N, 08028 Barcelona, Spain; 2 Paläontologie und Historische Geologie, Senckenberg Forschungsinstitut und Naturmuseum, Senckenberganlage 25, 60325 Frankfurt am Main, Germany; 3 Fundación Conjunto Paleontológico de Teruel-Dinópolis/Museo Aragonés de Paleontología, Av. Sagunto s/n, 44002 Teruel, Spain; 4 Instituto Geológico y Minero de España (Museo Geominero), c/ Cirilo Amorós 42, 46004 Valencia, Spain

**Keywords:** Ecology, Environmental sciences

## Abstract

Fossil records of vertebrate integuments are relatively common in both rocks, as compressions, and amber, as inclusions. The integument remains, mainly the Mesozoic ones, are of great interest due to the panoply of palaeobiological information they can provide. We describe two Spanish Cretaceous amber pieces that are of taphonomic importance, one bearing avian dinosaur feather remains and the other, mammalian hair. The preserved feather remains originated from an avian dinosaur resting in contact with a stalactite-shaped resin emission for the time it took for the fresh resin to harden. The second piece shows three hair strands recorded on a surface of desiccation, with the characteristic scale pattern exceptionally well preserved and the strands aligned together, which can be considered the record of a tuft. These assemblages were recorded through a rare biostratinomic process we call “pull off vestiture” that is different from the typical resin entrapment and embedding of organisms and biological remains, and unique to resins. The peculiarity of this process is supported by actualistic observations using sticky traps in Madagascar. Lastly, we reinterpret some exceptional records from the literature in the light of that process, thus bringing new insight to the taphonomic and palaeoecological understanding of the circumstances of their origins.

## Introduction

Amber is fossilised resin originating in ancient forests, with a high capacity for exceptional and three-dimensional preservation of biological remains, providing an outstanding source of information from past ecosystems^1^. Arthropods are the most common bioinclusions in amber^2,3^, although vertebrate remains are also often found, including those from Amphibia (e.g.,^4^) and crown group Reptilia (e.g.,^5^), such as body fossils and dinosaur feathers (e.g.,^6,7^). Dinosaur feathers and mammalian hair are keratin integumentary structures which constitute the different forms of vertebrate vestiture^8^. The term vestiture is used in this paper for the plumage and pelage of amniotes, thus excluding other dermal structures as scales or glands. Cretaceous amber is an important source of knowledge about feathers, in which they are particularly abundant, providing a panoply of palaeobiological evidence (e.g.,^6,7,9,10^). Recently, a new study has shown feather-like structures in pterosaurs, so these structures could appear in an archosaur ancestor of dinosaurs and pterosaurs or independently in these two groups^11^. Feathers are β-keratin integumentary structures^12^ which are present in avian and some non-avian dinosaurs and whose origin has been widely studied^13^. The feather structure is composed by medulla, cortex and cuticle (inner to outer)^14^. Feathers show high morphological variability, even in the same specimen, so their determination is challenging^14^.

Mammalian remains are rare in amber, and even more so in amber from the Cretaceous^15,16^. A partial mammalian skeleton which could correspond to a solenodontid was reported from the Miocene amber of the Dominican Republic^17^, but this is a unique finding since mammals are usually represented in amber by hairs, as in, for example, two records of abundant solenodontid-like hair also found in Dominican amber^18^. Hair is an α-keratin integumentary structure occurring since before the emergence of crown mammals^19,20^. Like feathers, the hair structure is composed of three layers; medulla, cortex and cuticle, going from innermost to outermost^21–23^. The hair cuticle consists of approximately rectangular, flattened, keratin scales composed of an exocuticle rich in sulphur and an endocuticle with low sulphur content^21,23^. The overlapping of the keratin scales provides the characteristic surface scale pattern (cuticular pattern) of mammalian hair^22,23^. The diverse hair surface scale patterns can provide information for the determination of mammalian taxa^18,22,23^. The scale pattern can differ between mammalian species, between individual strands of the same specimen, between stages of ontogenesis and along the length of a single strand depending on relative proximity to the basal region or the tip^21,24^. Furthermore, it is also possible for different species to show similar patterns^21,24^. The cuticular scale pattern is genetically controlled^23,25^. Its morphology and arrangement depend on biomechanical forces, which act on the cells during their hardening through the keratinisation process, and on growing speed^26^*.* Until now, the oldest described mammalian hair in amber corresponds to two strands from the late Albian French amber of Archingeay-Les Nouillers^16^. Mammalian hair is also reported from the early Cenomanian Burmese amber (northern Myanmar), even retaining the scale pattern^27^. One specimen of filamentous structure from the Santonian amber of Yantardakh (Taimyr, Russia) could also be a mammalian hair strand^15^. The youngest Cretaceous mammalian hair comes from the late Campanian amber of Grassy Lake in Canada^6^, but it is still undescribed. The record of mammalian hair in Cenozoic amber is comparatively richer (e.g., ^18,28,29^).

Based on its molecular composition, the preservation potential of feathers and hair is high^30^, although the keratin protein has a low fossilisation potential^10,31^. Dinosaur feathers from compression sites are well-known^30^, although the three-dimensional preservation in amber allows an exceptional visualisation of their anatomic structure^7^. Mammalian hair in Mesozoic mammaliaforms found in compression sites has been preserved as a halo of carbonised fur or tufts (e.g.,^19,32^), but usually the scale pattern is obscure or poorly preserved (e.g.,^33^). Furthermore, the taphonomic conditions that facilitated the preservation of vestitures in amber are little-known, and they have been usually related to resin flows produced near the forest floor (or ‘litter amber’ sensu^34^), requiring at least a contact of the individual before escaping^7^. In contrast, the taphonomic processes of insect preservation in amber have been widely studied^35,36^. Actualistic methods as sticky traps have been previously used to evaluate the accuracy of the record of forest arthropods in resin and amber^3,37^, but this kind of traps also records vertebrate vestitures and can provide an example of how resin traps these remains.

Here, we report on two Cretaceous amber pieces from Spain, containing a grouping of avian dinosaur feather remains and three strands of mammalian hair, respectively, resulting from the recording of vertebrate vestiture through a distinct biostratinomic process that we describe for the first time and that is unique to resins.

## Results

### Avian dinosaur feather remains

**Figure 1:**
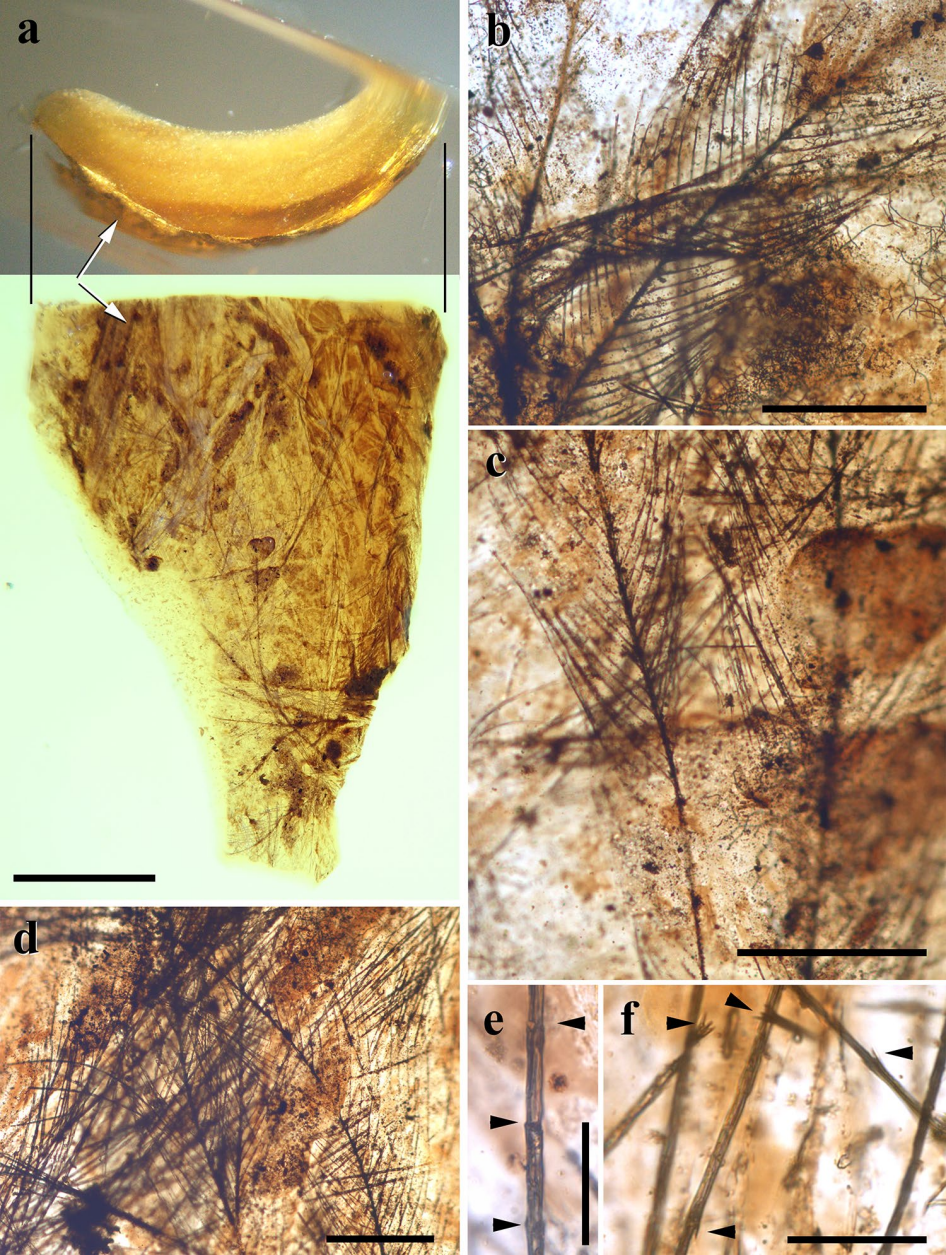
Avian dinosaur feather remains densely covering the convex surface of a flake fragment detached from a stalactite-shaped aerial amber piece (CPT-4200) found in the middle–earliest late Albian San Just outcrop (Utrillas, Teruel, Spain). (**a**) Two views of the stalactite-shaped aerial amber piece at the same scale showing the convex, external surface covered with feather remains (arrows: note that the surface is darkened, corresponding to a surface of desiccation). (**b–d**) Details of the dense, chaotic assemblage of feather remains (barbs) in the convex surface (note the left barb in (**c**) differentially preserved along its axis). (**e**, **f**) Details of some barbules showing the nodes of the pennulum (arrowheads in **e**), and spines in the internodes (arrowheads in **f**). Scale bars 1 mm (**a**), 0.2 mm (**b**–**d**), 0.05 mm (**e**, **f**). (**a**–**d**, **f**) are image compositions (Photoshop CS2, version 9.0; www.adobe.com).

A small flake (4.1 mm long and 2.8 mm at its greatest width) of an aerial, stalactite-shaped amber piece (CPT-4200, from San Just outcrop, Teruel, Spain) contains abundant feather remains (the barbs are easily recognised) of an avian dinosaur (Fig. 1). The flake piece is curved in cross section, with the convex (external) surface being dark and the concave one being of the same colour and transparency as the rest of the piece (Fig. 1a). These features indicate that it is a fragment of one of the concentric layers, originating from a resin flow; both the internal core of the piece and a potential later layer (or layers) were lost. The external surface is darker due to original desiccation under aerial conditions. This surface is completely covered by feather barbs dispersed at random without preferential orientation. The barbs (at least a few millimetres long and ca. 0.29 mm wide, with barbules ca. 0.26 mm long; Fig. 1a–d) are not impressions in the surface, but conserved original organic matter, although some small patches are missing (Fig. 1c). Barbs and barbules are well preserved, including the nodes and internodes of the pennula (Fig. 1e,f). The sticky external resin layer must have already been somewhat hardened when the feathers came into contact with it because it does not show deformation from contact and the barbules did not penetrate the resin but are present over the whole original convex surface, as preserved.

### Mammalian hair strands

**Figure 2:**
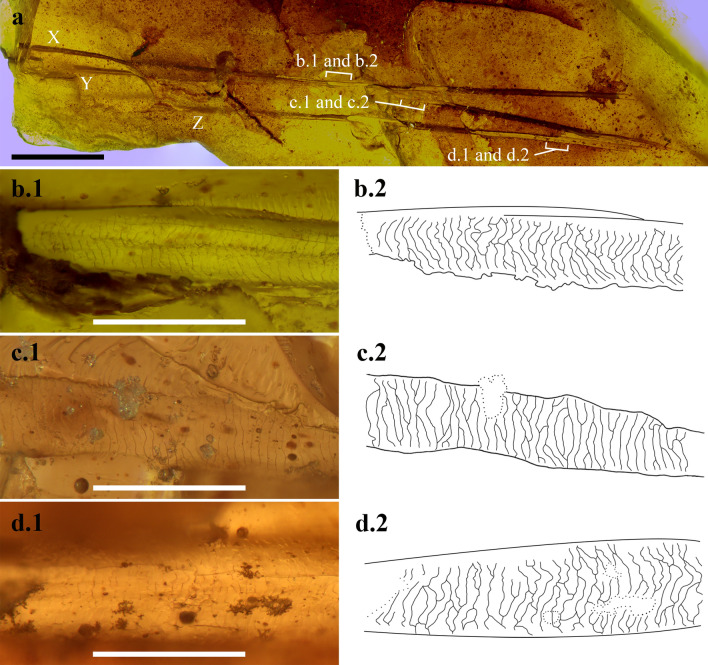
Hair strands aligned in the amber fragment AR-1-A-2019.88.1. (Ariño, Teruel, Spain), dated as early Albian. (**a**) Image composition of the general view of the three strands named X, Y and Z, indicating the locations of the following images and drawings. (**b–d**) Superficial scale patterns as cast prints in the amber, illustrated as micrographs and camera lucida drawings from X, Y and Z strands, respectively, all at the same scale. Scale bars 1 mm (**a**), 0.1 mm (**b**–**d**). Image composition and drawings prepared with Photoshop CS6, version 13.0 (www.adobe.com).

**Figure 3:**
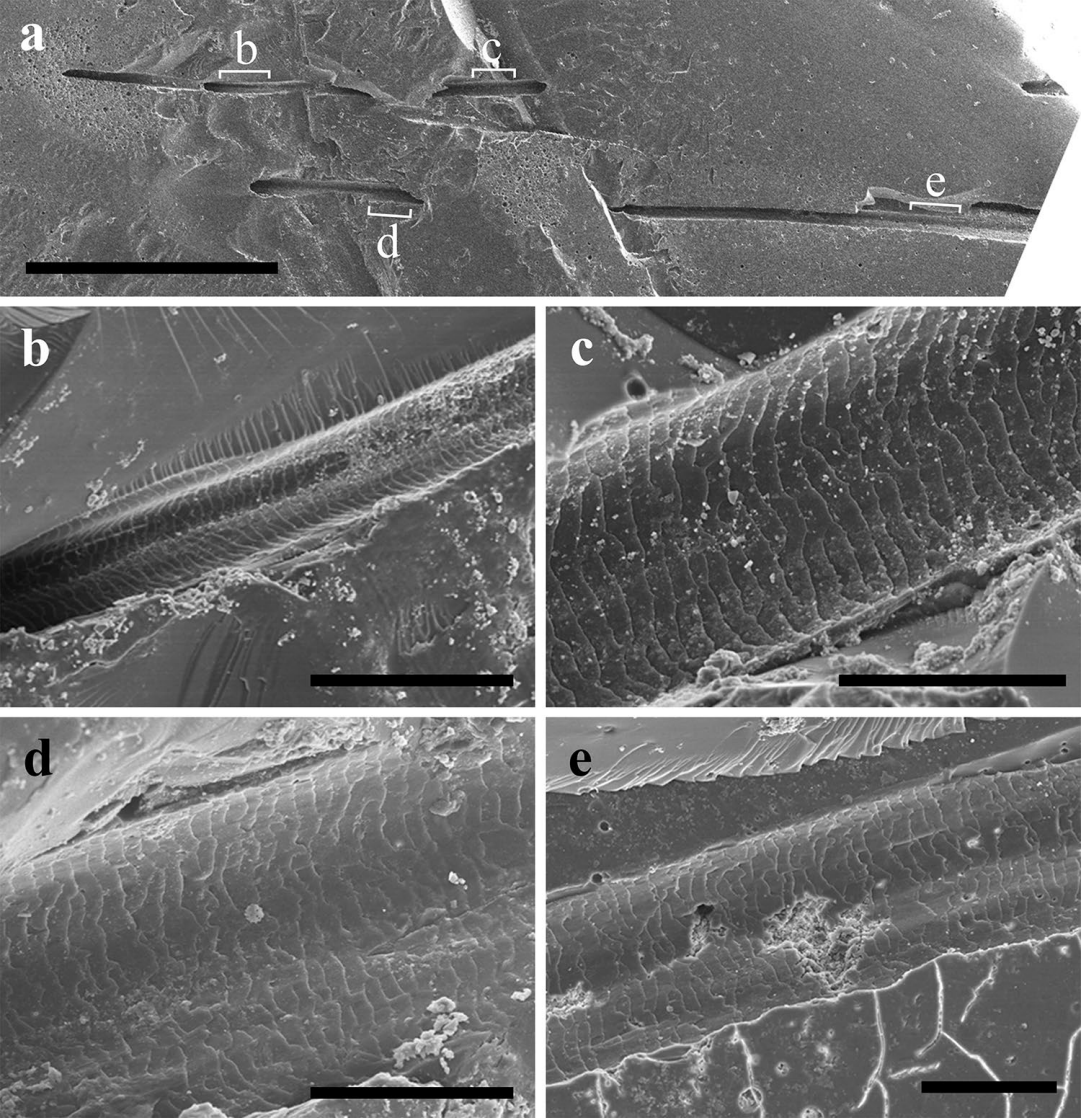
SEM images of the scale pattern of hair strands as cast prints in the amber fragment AR-1-A-2019.88.1 (Ariño, Teruel, Spain), dated as early Albian (see Fig. 2). (**a**) General view of the amber surface and the hair strands, indicating the locations of the following images. (**b**, **c**) Scale patterns of the hair strand X. (**d**, **e**) Scale patterns of the hair strand Z. Scale bars 1 mm (**a**), 0.1 mm (**b**), 0.05 mm (**c**–**e**).

Three mostly parallel-aligned strands of mammalian hair are present in the fragment AR-1-A-2019.88.1 (from Ariño outcrop, Teruel, Spain) (Figs. 2a, S1) (see the description of the whole amber piece AR-1-A-2019.88 in the Supplementary information). They are named X, Y and Z and are exceptionally well preserved, even showing the surface scale pattern (Fig. 2b–d). The scale pattern is easily observed due to the piece having been broken during its extraction, which has facilitated its drawing and SEM imaging (Fig. 3), in contrast to the two hair strands in the French amber, which are poorly preserved^16^, and the hair records found in compression sites (e.g.,^19,33^). The three hair strands are brown and straight, and apparently they were rigid. Strand Y is broken, and it is possible that the two fragments are actually from two different strands. There is a discontinuous gap between them where the amber piece seems to be broken, but as they are in the same plane below strand X, we treat them as two fragments of the same strand. The strands are incomplete, and the tips and hair follicles are not preserved, so the orientation of the scale arrangement is not possible to determine. The scale pattern can be partly seen in the external brown surface, but the transparency of the amber fragment facilitates visualisation of the scale pattern mainly as impressions (or cast prints) in the amber, as shown on the internal surfaces of the broken sections. Strand X is 6.72 mm long and around 0.07 mm in diameter; broken strand Y is composed of two strand fragments which are 3.26 mm and 3.20 mm long, respectively, with a diameter of around 0.07 mm, and strand Z is 6.19 mm long and around 0.07 mm in diameter. The cross section of the hair strands cannot be visualised, so medulla and cortex cannot be seen, and are possibly not preserved along the strands, although they could be in those parts where the brown surface of the cuticle is present. An accurate description of the scale pattern was achieved based on the clearly marked scale margins appearing as impressions in the amber.

The scale pattern and proportions of the three hair strands are similar. Scales are wider than they are long and are in a transverse position in relation to the hair longitudinal axis (Figs. 2b–d, 3). Scale length varies between 5–7 µm. The surface scale pattern is wavy in all three strands, mostly regular in strand Y and slightly irregular in strands X and Z. Scale margins are smooth and close together. Following the terminology of Chernova^23^, the cuticular pattern corresponds to a non-annular morphotype, adhering and tegular, with more than one scale embracing the shaft, to which they are strongly attached, and clearly imbricate.

### Actualistic data

**Figure 4:**
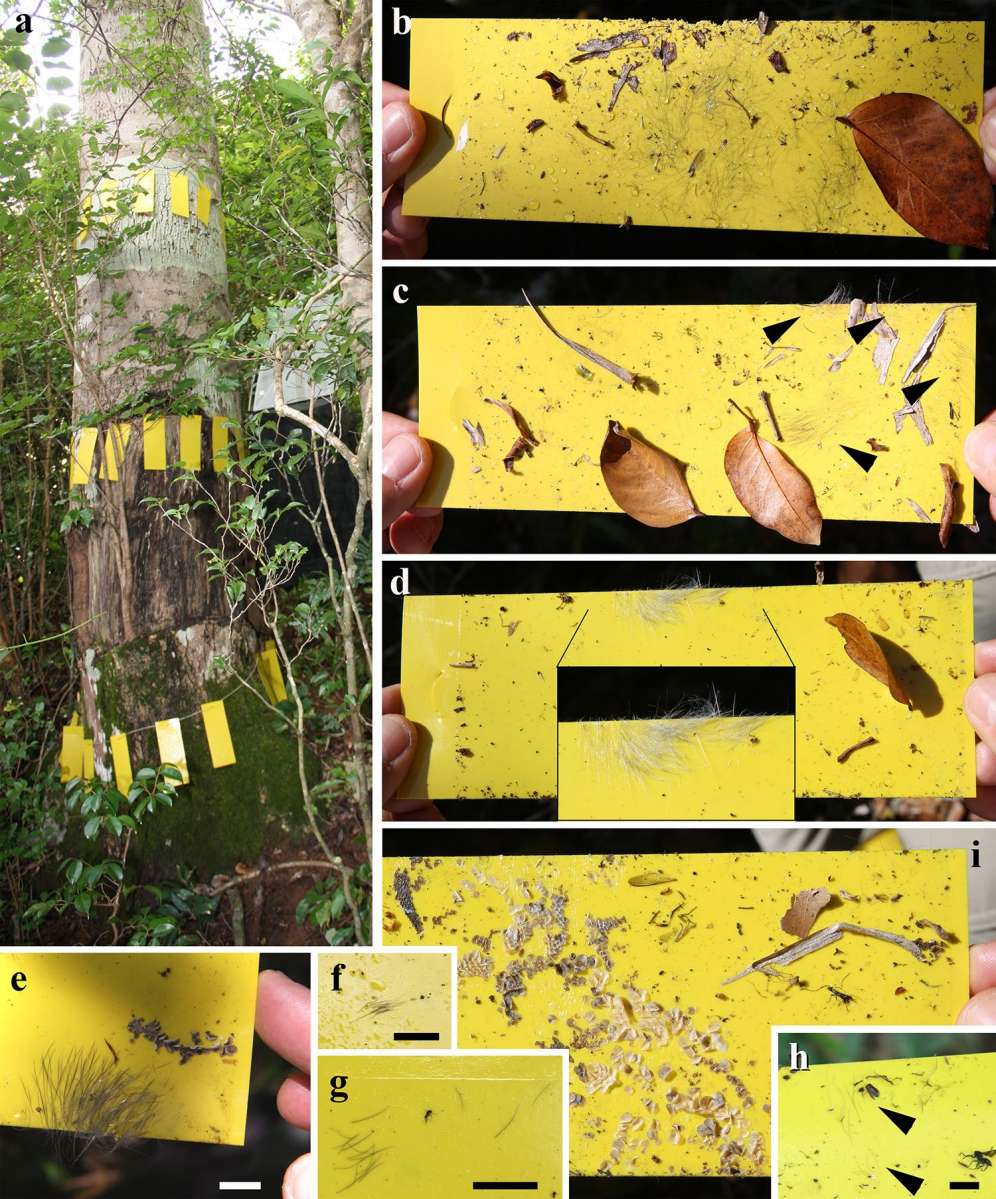
Examples of non-natural “pull off vestiture” process in actuotaphonomic research in Madagascar using yellow sticky traps on trunks of the resiniferous tree species *Hymenaea verrucosa* (Fabaceae) and *Canarium madagascariense* (Burseraceae). (**a**) Three sticky trap lines at 0, 1 and 2 m height, respectively, in *H*. *verrucosa*, in Sacaramy area (Antsiranana), tree 2H2 (2R2), campaign 2015. (**b–d**) Sticky traps with abundant trapped hair, in *H*. *verrucosa* tree 2, at 1 m, 2 m and 0 m heights, respectively, in Ambahy community (Nosy Varika, Mananjary region), campaign 2013 (see arrowheads in **c** indicating tufts and inset in **d** showing the tuft enlarged). (**e**) Detail of tuft and small portion of lizard skin in sticky trap of *H*. *verrucosa* tree 1 at 1 m height, Ambahy community, campaign 2013. (**f**) *H*. *verrucosa* tree 2H1 (2R1) at 2 m height, Sacaramy area, campaign 2015. (**g**) *H*. *verrucosa* tree 3R5 (3H4) at 1 m height, Analamandrofo forest in Andranotsara, at 40 km south of Sambava city, campaign 2017. (**h**) Hair (arrowheads), most likely of the abundant lemurs in the canopy of the research area, sticky trap in *C*. *madagascariense* tree 3 at 2 m height, Ranomafana National Park, campaign 2013. (**i**) Sticky trap with abundant lizard skin (geckos) from *H*. *verrucosa* tree 2 at 0 m height, Ambahy community, campaign 2013. Sticky traps measure ca. 20 × 7.5 cm. Scale bars 1 cm (**e**–**h**).

**Figure 5:**
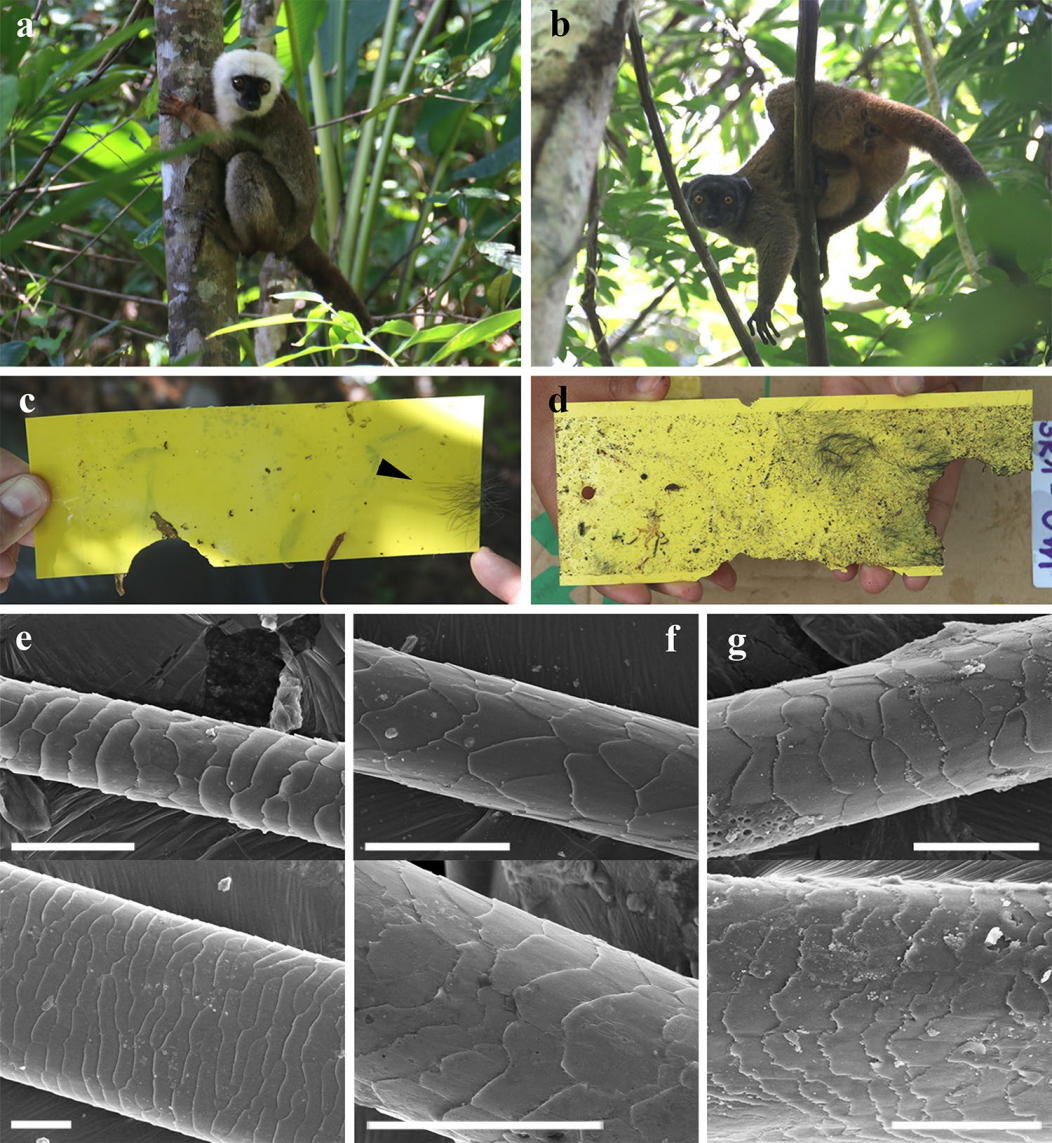
Images from actualistic data obtained in Malagasy forests. (**a**, **b**) Two lemur species in the tree canopy in the Ranomafana National Park. (**c**, **d**) Sticky traps with adhered hair tufts (indicated with an arrowhead in **c**) and gnaw marks, from *Hymenaea verrucosa* tree 1 at 2 m height, in Ambahy community (Nosy Varika, Mananjary region), campaign 2013, and *H*. *verrucosa* tree 3R1 at 0 m height, in Analamandrofo forest in Andranotsara, at 40 km south of Sambava city, campaign 2017, respectively. (**e–g**) SEM images of hair strands from *H*. *verrucosa* tree 2 at 0 m height, most likely of a rodent, in Ambahy community, campaign 2013 (sticky trap from Fig. 4d), *H*. *verrucosa* tree 1 at 1 m height, Ambahy community, campaign 2013 (sticky trap from Fig. 4e), and *Canarium madagascariense* tree 3 at 2 m height, most likely of a lemur, Ranomafana National Park, campaign 2013 (sticky trap from Fig. 4h), respectively. Sticky traps measure ca. 20 × 7.5 cm. Scale bars 0.02 mm (**e**), 0.04 mm (**f**), 0.03 mm (**g**).

Mammalian hair was recorded in sticky traps located at different heights in the trunk of the angiosperms *Hymenaea verrucosa* Gaertner, 1791 and *Canarium madagascariense* Engler, 1883 in Madagascar (Figs. 4, 5). Isolated strands are found in several sticky traps, although even tufts are present in others. Mammalian hair corresponds to the 0.11% and 0.01% of the total remains adhered to sticky traps in *H*. *verrucosa* and *C*. *madagascariense*, respectively. The strands were pulled off retaining the proximal and distal portions. Sticky traps located at 0 m high in the trees recorded hair strands and tufts of rodents that are most likely non-arboreal (Fig. 5e); several of these sticky traps were even gnawed (Fig. 5c,d). Hair, most likely from lemurs, is found in sticky traps at 2 m high in *C*. *madagascariense* (Figs. 4h, 5g). In contrast, feathers are not found adhered to the traps.

## Discussion

**Figure 6:**
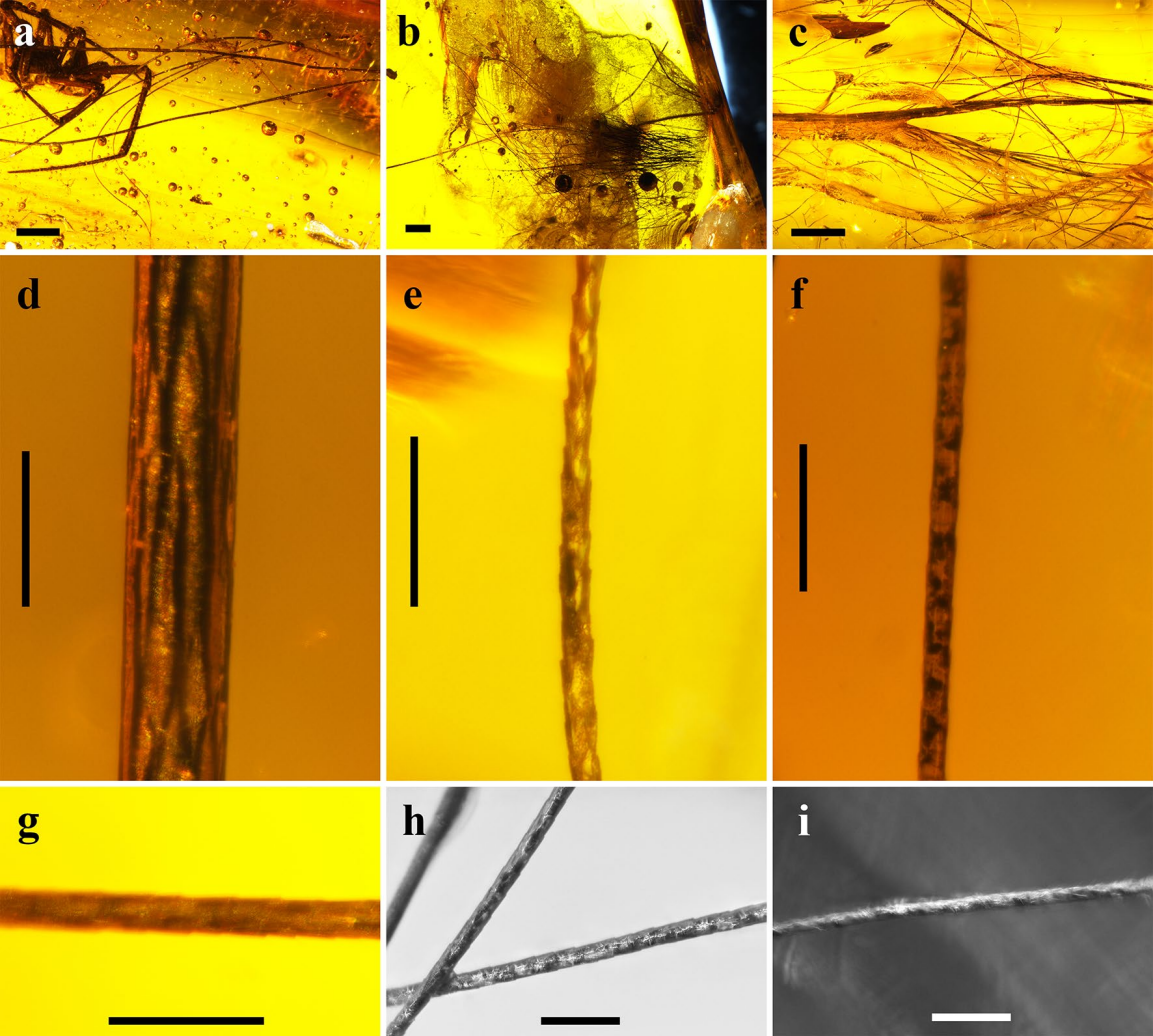
Mammalian hair in Eocene Baltic amber. (**a–c**) Tufts in pieces SMF-Be-5160 (showing a spider as syninclusion), SMF-Be-8362 and SMF-Be-365, respectively. (**d–i**) Details of hair strands in pieces SMF-Be-5160, SMF-Be-365, SMF-Be-8362, SMF-Be-5160, SMF-Be-365 and SMF-Be-5161, respectively. Scale bars 1 mm (**a**–**c**), 0.1 mm (**d**–**i**).

Most isolated feathers found in Burmese amber have been determined as belonging to Enantiornithes based on the presence of skeletal remains with associated feathers of this group (e.g., 38), although this hypothesis is controversial due to the many feather morphotypes that are not associated with skeletal remains^7^. The San Just feather remains could be consistent with this taxonomic affinity, but the absence of enantiornithine skeletal records in Spanish amber precludes even a tentative determination. Filamentous elongated remains are often found in amber and they can be confused with hair strands. Fungal mycelia have been reported in ambers from different deposits, showing hyphae of 2–8 μm diameter (e.g.,^39,40^), as opposed to the Ariño strands which are around 70 μm in diameter. Undetermined plant fibres also appear in amber, but they are usually distinct from hair-like structures, as they can be very irregular in shape. The key characteristic of hair strands is their scale pattern^22,23^, although Chernova^14^ noticed a similarity between the microstructures of determined hairs and feathers as a result of morphological convergence. The Ariño strands clearly correspond to mammalian hair as their scale pattern is very similar to others from fossil (Mesozoic and Cenozoic) and extant hair specimens (Figs. 5e–g, 6).

**Figure 7:**
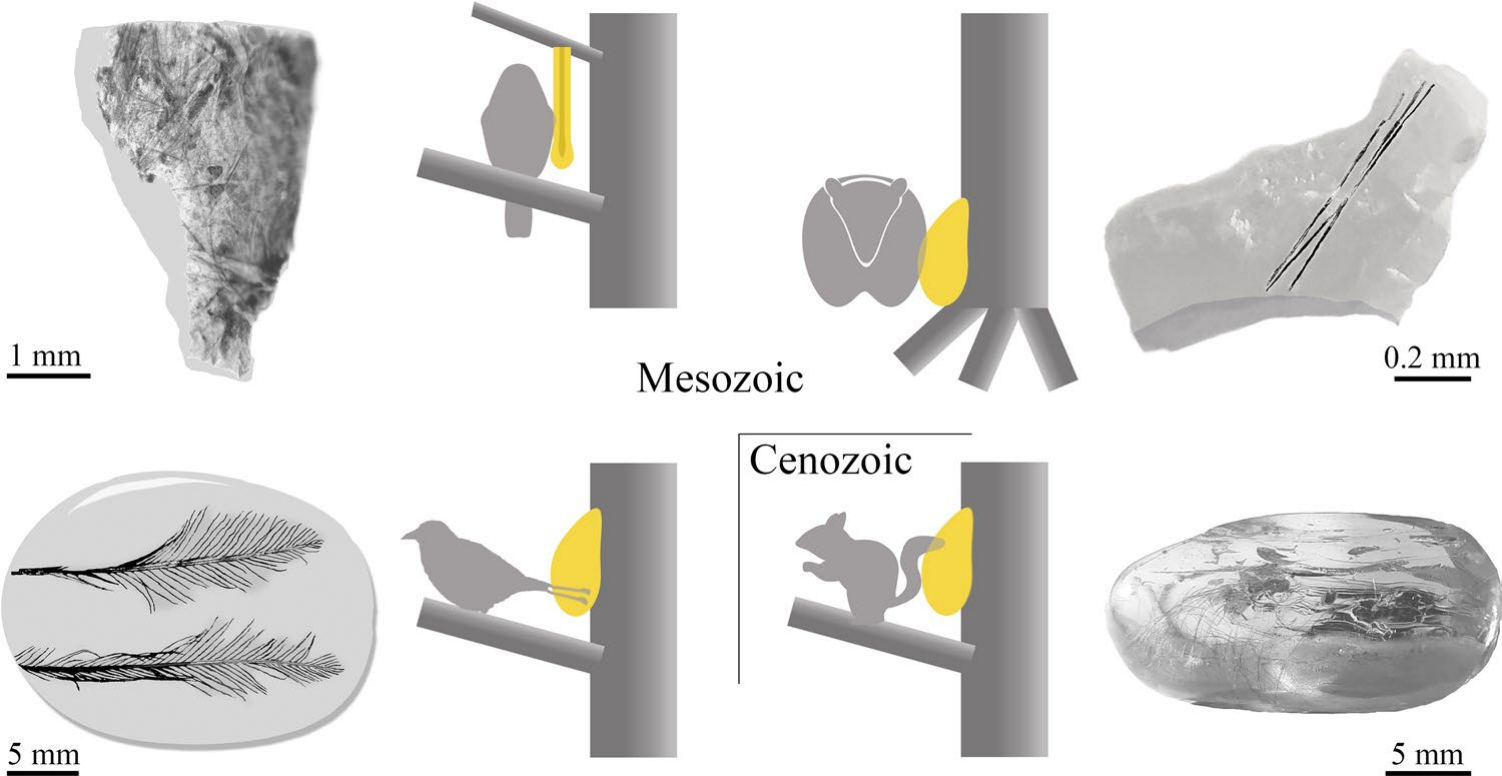
Schema of the two examples studied and another two from the literature of the “pull off vestiture” process as interpreted. From upper left to lower right: Spanish pieces CPT-4200, containing feather remains and AR-1-A-2019.88.1 containing hair tuft of an indeterminate mammal, Burmese piece DIP-V-17194 (modified from^48^) containing a pair of feathers from tail plumage, most likely with ornamental function, and Baltic piece SMF-Be-2009 containing mammalian hair. Illustration created using Adobe Photoshop CS2, version 9.0 (www.adobe.com).

Accurate determination of isolated hair in deep time is especially difficult, if not impossible. In the Early Cretaceous Spanish localities of the Galve area, near Ariño, mammalian remains belonging to Dryolestida, Multituberculata (Eobaataridae, Paulchoffatiidae, Pinheirodontidae, Plagiaulacidae/Eobaataridae), Symmetrodonta (Spalacotheriidae) and the Peramuridae family have been identified^41^; these taxa are extinct and information about their hair scale pattern is obscure. An exceptionally well-preserved specimen from the late Barremian Las Hoyas locality (Cuenca, Spain), identified as *Spinolestes xenarthrosus* Martin et al. 2015 (Eutriconodonta: Gobiconodontidae) has provided important information about the eutriconodont fauna, including integumentary structures, as this specimen has preserved the hair scale pattern^33^. Hair strands of *S*. *xenarthrosus* include two morphotypes of scale patterns, as the primary hairs show imbricate, ovate scales forming an irregular mosaic and the secondary hairs possess annular scales embracing the shaft with simple or serrated free margins (extended data Fig. 5 in^33^). The scale pattern of the Ariño hair strands does not correspond to the morphotypes of the Las Hoyas specimen, as more than one scale embraces the shaft and the pattern is wavy with smooth margins. Considering extant taxa, the scale pattern of mustelids (Carnivora: Mustelidae) is very similar to that of the Ariño hair strands, for example, *Martes martes* Linnaeus, 1758 (plate 72–80 in^22^) and the wavy pattern at the pars basalis of *Lutra lutra* Linnaeus, 1958 (Fig. 7 in^42^). Despite all the above knowledge, accurate determination of the hair strands from Ariño is not also possible, due to the poorly preserved scale pattern of fossil hair in Cretaceous mammalian specimens. The Ariño hair strands from the early Albian are the oldest mammalian record in amber and increase the previous known Cretaceous record of mammalian hair confined to late Albian–early Cenomanian ambers^16,27^. New descriptions of scale patterns of hair in Cretaceous amber, whose preservation capacity is exceptionally high, as seen in the Ariño hair strands, could shed further light on the evolution of this mammalian character through time.

Based on the matching scale pattern and proportions of the three hair strands, their proximity and alignment in the amber piece, we assume that they were a tuft. Although they are only three strands, their disposition and the lateral similarity in diameter and scale pattern support our inference that they are not randomly detached strands. This is a unique record in the Cretaceous, as other hair strands in amber (France and Myanmar) appear as isolated specimens^16,27^. The two hair strands in French amber are in the same piece, separated by only 1 mm, but they cannot be confirmed as a tuft because of their poor preservation^16^. Therefore, the Ariño hair strands show more similarities with the record of tufts in the Eocene Baltic amber (e.g.,^28,29^) (Fig. 6).

Both amber pieces presented in this study correspond to resin flows exposed to aerial conditions for a time, based on their morphology and the presence of surfaces of desiccation. Despite the small size of the amber piece CPT-4200 (showing feather remains), the manner in which the barbs cover the whole surface of desiccation, as preserved, indicates that this record was not the consequence of contact between a detached feather, or feathers, and the sticky resin emission. This record can best be explained thus: an avian dinosaur made contact with the resin emission, resting there for the necessary amount of time, for example during sleep, for the more external barbs of its vestiture to become firmly fixed in the hardened resin. Later, with the movement of the animal, the fixed barbs detached. However, it can be observed that some portions of the barbs were apparently better fixed than others as there is differential preservation along the barbs (Fig. 1c). Regarding the amber fragment AR-1-A-2019.88.1 (showing the hair tuft), the hair strands could have become embedded slowly while the mammal in question was resting or sleeping near the resin source, in similar fashion to the feathers in piece CPT-4200.

The two amber records described herein are very peculiar and their origins are far from the common process for generation of bioinclusions in resins that are preserved as amber or copal. Typically, a bioinclusion originates when an animal or a detached organismal remnant makes contact with fresh, sticky resin and is thus trapped and then embedded. Here, we present the “pull off vestiture” biostratinomic process unique to resins, which we define as follows: “the entrapment of external portions of vertebrate vestiture, more specifically small portions of plumage and pelage, of living individuals that had rested for a time in contact with a fresh, sticky resin emission that hardened and retained those vestiture portions”. Note that this process does not imply the death of the animal. The process requires a type of resin that can harden very quickly after being exuded. Such a feature has been observed in extant resins from *Agathis australis* (D. Don) Loudon, 1829 (gymnosperm) in New Zealand and *Hymenaea verrucosa* in Madagascar, but not under all conditions (own observations). Furthermore, actualistic observations using sticky traps in *H*. *verrucosa* and *Canarium madagascariense* in Madagascar support this hypothesis (Figs. 4, 5). These records are similar to those of the natural “pull off vestiture” process of resins, although the trapping capacity of the sticky traps is much higher. Hair would be trapped immediately in the sticky traps with minimal contact, whereas the “pull off vestiture” process involving resin requires contact to be sustained for the time it takes for the resin to harden. Squamate reptile scaly skin was also recorded on the sticky traps (Fig. 4i), as has been found in Cretaceous amber from France, Lebanon and Myanmar^43–46^, but these kinds of record would be recorded differently as they could be explained by detached portions of lizard moults which do not require high stickiness.

The “pull off vestiture” biostratinomic process described herein is unique to resins and clarifies some intriguing findings in Cretaceous and Cenozoic ambers. Other semi-fluid preservative materials, like asphalt (highly viscous liquid or a semi-solid form of petroleum) or tar seeps, do not pull off vertebrate vestiture while allowing the survival of the individual^47^. Paired ornamental feathers of enantiornithine tails in the manner of their distal parts in relative live position (each pair) from the Burmese amber record have been reported^48,49^. Xing et al.^48^ commented that the paired feathers, which they found abundantly, could easily be removed, maybe as a sacrificial gesture in defensive behaviour. The record of these abundant findings could be related to the “pull off vestiture” process, as are other enantiornithine feather records in amber (e.g.,^7^). The tail feathers could become stuck in the resin while the individual was resting or sleeping close to the resin source in the tree, and after a time, when the resin was hardened and the individual left, the feathers were removed, similar to the biostratinomic process of the San Just and Ariño records in this work (Fig. 7). The tufts found in Eocene Baltic amber only preserve the distal parts of the strands^29^ and are coincident with the “pull off vestiture” process (Fig. 7). The basal parts of the hairs (including the follicles) are not preserved in the amber pieces because typically only the distal parts contacted and became embedded in the resin emissions to undergo the “pull off vestiture” process. Records of mammalian hair strands in Miocene Dominican amber correspond to isolated strands with different orientations, not forming tufts, and even retaining basal parts (e.g.,^18,50^), so they could have been blown by the wind or shed randomly by the mammalian individual and do not correspond to a “pull off vestiture” process. The rich record of mammalian tufts in Baltic amber could be related to the presence of arboreal mammals such as sciuromorph rodents^51^, although Sidorchuk et al.^29^ proposed the floor-dwellers, erinaceomorph amphilemurids. The extreme scarcity of hair in Cretaceous amber suggests that the arboreal mammals did not inhabit the resiniferous forests during this period, although an arboreal lifestyle has been suggested for gliding Mesozoic mammals from compression sites (e.g.,^32,52^). The new biostratinomic approach described herein can explain records of vertebrates in amber and provide new inferences of palaeoecological information about the ancient resiniferous forests.

## Material and methods

The San Just outcrop is located near Utrillas (Teruel, Spain) and corresponds geologically to the Middle or Upper Member of the Escucha Formation^53,54^. It has been dated as middle–earliest late Albian based on palynological data, and related to a swamp plain environment^55^. It is the type-locality of 23 species and its diverse record of bioinclusions contains coprolites, fungi, plants, arachnids, 12 insect orders and dinosaur feathers^53,54^. More data about the amber piece CPT-4200 are available in the Supplementary information.

The Ariño amber outcrop is in the Santa María opencast mine near Ariño (Teruel, Spain), which has provided a diverse fossil record from the bonebed layer AR-1, including coprolites, algae, plants, molluscs, ostracods, fish, turtles, crocodiles and dinosaurs^56^, and is the type-locality of nine new taxa to date. This layer, belonging to the Middle Member of the Escucha Formation, has been dated as early Albian based on the charophyte assemblage^57^ and related to a freshwater swamp plain in a deltaic-estuarine system with salinity variations due to marine inputs under subtropical-tropical climate conditions^58^. The amber piece AR-1-A-2019.88 with the mammalian hair studied herein was found in AR-1, composed of marls within an alternation of marl and limestone levels with alkaline oligotrophic origin, pond or shallow lake, and was unearthed from the layer in separate fragments; a description of the piece is available in the Supplementary information (Fig. S1).

The San Just piece CPT-4200 was photographed with a digital camera Olympus Camedia MODEL N.C5050 ZOOM attached to an Olympus SZX9 stereomicroscope, and the detailed micrographs of the barbules, using a ColorView IIIu Soft Imaging System attached to an Olympus BX51 compound microscope; the frontal view of the feather remains (Fig. 1a) was taken using a digital camera Canon EOS 650D and z-stacked automatically by the software Macrofotografía version 1.1.0.5 (www.macrorail.com). A compound microscope Olympus CX41 equipped with an attached camera lucida tube and a digital camera sCMEX-20 was used to make the drawings and the micrographs of the mammalian hair strands and other syninclusions in piece AR-1-A-2019.88; these micrographs were processed with ImageFocusAlpha version 1.3.7.12967.20180920 (www.euromex.com). SEM images of the fossil and extant hair strands with gold sputtering were obtained with a Hitachi S4800 Electronic Microscope at the Microscopy Services (SCSIE) of the University of Valencia (Spain). Figures were prepared with Photoshop CS2 version 9.0 (www.adobe.com) and Photoshop CS6 version 13.0 (www.adobe.com). The description of the scale pattern of the hair strands follows the nomenclature of Teerink^22^ and Chernova^23^.

Regarding the Baltic amber pieces (Fig. 6), they are housed at the Senckenberg Research Institute and Natural History Museum (SMF) in Frankfurt (Germany). The colour photographs and Z-stacks images were performed under a Nikon SMZ25 microscope, using Nikon SHR Plan Apo 0.5x and SHR Plan Apo 2x objectives with a microscope camera Nikon DS-Ri2 and the NIS-Element software version 4.51.00 (www.microscope.healthcare.nikon.com). Infrared reflected photomicrographs (black and white) were taken with a Nikon Eclipse ME600D. All these methods were undertaken at the SMF.

The actualistic data were obtained in Madagascar with sticky traps following the methodology of Solórzano Kraemer et al.^3^. The sticky traps were located around 12 trees of the species *Hymenaea verrucosa* (Fabaceae) and four trees of the species *Canarium madagascariense* (Burseraceae) at 0, 1 and 2 m high during eight days in Sacaramy area (Antsiranana), Ambahy community (Nosy Varika, Mananjary region), Analamandrofo forest, in Andranotsara at 40 km south of Sambava city and Ranomafana National Park, during the 2013, 2015 and 2017 campaigns^59^ and were photographed with a Canon EOS 40D digital camera. *Hymenaea* is a genus of resiniferous tree that originated amber deposits in several places such as Mexico and Dominican Republic. Nowadays, representatives of this genus are found in Madagascar, where an actuotaphonomic work was addressed to study how the resin traps biological remains using sticky traps. The resiniferous genus *Canarium* occurs in Malagasy forests with dissimilar environmental conditions, thus allowing the comparison of resin trapping in different ecosystems.

## Supplementary information

Supplementary 1.

## Data Availability

All data that support this study are available in the main text or in the Supplementary information. Institutions that host the Spanish amber pieces are indicated in the Supplementary information. Actualistic data from sticky traps are available at the SMF in Frankfurt (Germany).
