# Supplementary material for: Cretaceous amniote integuments recorded through a taphonomic process unique to resins
Source: Sci Rep. 2020 Nov 16;10:19840. doi: 10.1038/s41598-020-76830-8 (PMC7669849; doi:10.1038/s41598-020-76830-8)
Supplement: Supplementary file 1 — Supplementary 1. [file 41598_2020_76830_MOESM1_ESM.pdf]

**Supplementary information**

# Cretaceous amniote integuments recorded through a taphonomic process unique to resins

Sergio Álvarez-Parra*, Xavier Delclòs, Mónica M. Solórzano-Kraemer, Luis Alcalá & Enrique Peñalver

*sergio.alvarez-parra@ub.edu

## Supplementary material and methods

### *Amber piece CPT-4200*

The small amber flake is embedded in a prism of epoxy resin important for the preservation of amber pieces with bioinclusions (*60*). It was found during an excavation in the San Just amber-bearing outcrop near Utrillas (Teruel, Spain) in 2007. It is housed at the Museo Aragonés de Paleontología (Fundación Conjunto Paleontológico de Teruel-Dinópolis, Teruel) with the accession number CPT-4200. Its primary field number was SJ-07-147.

### *Amber piece AR-1-A-2019.88*

The piece was found during an excavation in the Santa María opencast mine near Ariño (Teruel) in 2019 and is housed at the Museo Aragonés de Paleontología (Fundación Conjunto Paleontológico de Teruel-Dinópolis, Teruel). It is made up of seven fragments (AR-1-A-2019.88.1 to 88.7). Three strands of mammalian hair are in one of these fragments (AR-1-A-2019.88.1) and the other two bioinclusions (syninclusions) are in two different fragments, one being a partial undetermined insect antenna (AR-1-A-2019.88.2) and the other an undetermined arthropod (AR-1-A-2019.88.3) (Fig. S1a). Four of these fragments are less than 1 mm long and do not include visible fossil remains. The fossiliferous fragments AR-1-A-2019.88.1 and AR-1-A-2019.88.2 are around 1 mm long, and AR-1-A-2019.88.3 is 1.45 mm long. None are embedded in epoxy resin. The amber is brownish-yellow with abundant dark microscopic inclusions corresponding to double emulsions of phloem sap typically present in Cretaceous ambers (*61*), so these characteristics give a slightly opaque aspect to the fragments, mostly in the case of AR-1-A-2019.88.3.

Fragment AR-1-A-2019.88.2 includes a partial insect antenna (Fig. S1b). It corresponds to a filiform morphotype, with four setose and long antennomeres, maybe of the apical section. Each antennomere is 0.30 mm long (although one of them seems to be incomplete and is 0.26 mm long) and 0.06 mm wide. This type of antenna could correspond to Blattodea (*62*), but an accurate determination is not possible because of the poor and partial preservation. This fragment shows a surface of desiccation (Fig. S1c), indicating that the resin was under aerial conditions for a time in the resiniferous tree, but there is no evidence from which to infer an approximate height. It was possibly close to the litter.

Fragment AR-1-A-2019.88.3 is very opaque with abundant double emulsions of sap. An undetermined fossil remnant shows in an edge of the fragment (Fig. S1d). It could be a partial body remain of an arthropod, but its poor preservation prevents identification. An emerging long structure could correspond to an antenna.

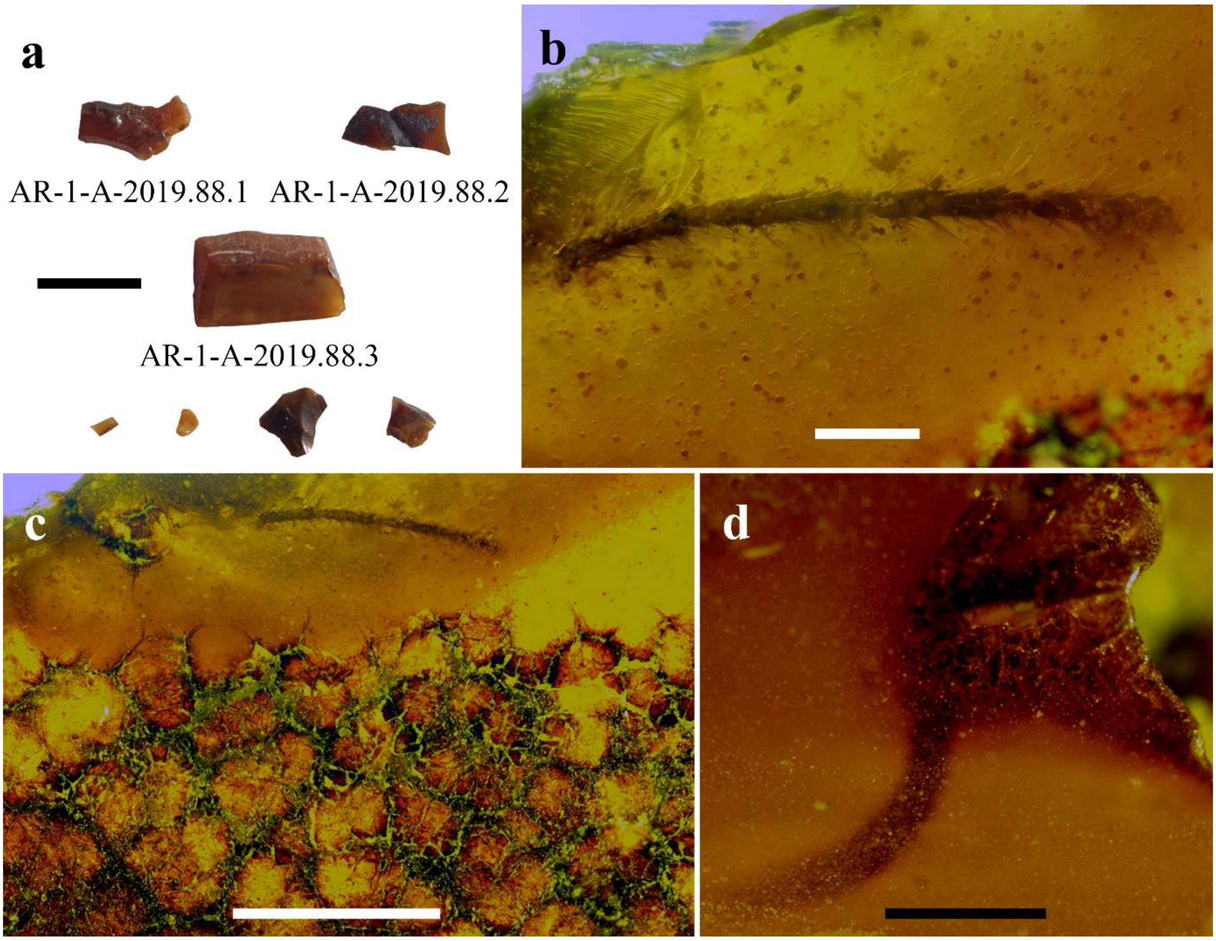


**Supplementary Figure S1.** Fragmented amber piece AR-1-A-2019.88 from Ariño outcrop (Teruel, Spain), dated as early Albian. (**a**) All the fragments at the same scale. (**b**) Insect antenna in AR-1-A-2019.88.2. (**c**) Surface of desiccation in AR-1-A-2019.88.2. (**d**) Image composition of an arthropod in AR-1-A-2019.88.3. Scale bars 1 mm (a, c, d), 0.2 mm (b).

**References (Supplementary information)**

60. Nascimbene, P. & Silverstein, H. The preparation of fragile Cretaceous ambers for conservation and study of organismal inclusions. In *Studies on Fossils in Amber, with Particular Reference to the Cretaceous of New Jersey* (ed. Grimaldi, D.) 93–102 (Backhuys Publishers, Leiden, 2000).

61. Lozano, R. P. *et al*. Phloem sap in Cretaceous ambers as abundant double emulsions preserving organic and inorganic residues. *Sci. Rep.* **10,** 9751 (2020).

62. Bell, W. J., Roth, L. M. & Nalepa, C. A. Eds, *Cockroaches: ecology, behavior, and natural history*. (The Johns Hopkins University Press, Baltimore, 2007).
